# Notes and News

**Published:** 1895-10-19

**Authors:** 


### NOTES AND NEWS.

(Collected and Communicated.)

The establishment of public baths in New York is being pushed vigorously forward. Plans have already been prepared and approved, and the baths are expected to be ready for use by the beginning of next summer.

In spite of exposures of fraud in connection with the "Lourdes miracles" that might well have shaken the faith of believers, the pilgrimage has been larger than ever this year. From fifteen to twenty thousand patients were received every day. Statistics are not forthcoming as to how many pilgrims never returned to their homes, but it is remarked that the journey of returning pilgrims was marked with fewer harrowing scenes than that of those arriving.

We have much pleasure in announcing that Mr. D. J. MacRae, the editor of *The Financial Times*, has distributed £2,000 amongst the following charities through Messrs. Michael Abrahams, Sons, and Co.:—

| | |
|---|---|
| University College Hospital ... ... ... | £250 |
| All Saints' Convalescent Home, Eastbourne | 100 |
| Westminster Hospital ... ... ... ... | 100 |
| Nurses' Pension Fund ... ... ... ... | 100 |
| Dreadnought Seamen's Hospital ... ... | 100 |
| Brompton Hospital ... ... ... ... | 100 |
| Cancer Hospital... ... ... ... ... | 100 |
| East London Hospital... ... ... ... | 100 |
| The National Hospital for the Paralysed ... | 100 |
| Jewish Board of Guardians ... ... ... | 100 |
| Jewish Hospital... ... ... ... ... | 100 |
| Jewish Free School ... ... ... ... | 100 |
| Jewish Blind Society ... ... ... ... | 100 |
| Westminster Jewish Free School ... ... | 50 |
| Deaf and Dumb Asylum ... ... ... | 50 |
| Reedham Institute for Homeless Boys ... | 100 |
| St. Anne's Home, Redhill ... ... ... | 100 |
| Rev. Hobbs (Bewdley), and another... ... | 250 |

We welcome the interest thus displayed by our contemporary in the progress of hospitals and kindred charities, and hope that its example may be followed by others in the near future.

Dr. Paquin, Professor of Bacteriology, Missouri University, is using the serum of horses for the cure of tuberculosis. He considers his experiments as satisfactory in confirming its usefulness.

By the will of the late Mrs. Fraser, widow of Dr. James Fraser, second Bishop of Manchester, large sums of money are to be divided between charities, many and various. Manchester hospitals and institutions naturally head the list, amongst which to the Cancer Hospital, Stanley Grove, is allotted £2,000; a special cot is endowed in the Ardwick and Ancoats Hospital; and £1,000 is left to the Manchester Royal Infirmary. To the Radcliffe Infirmary, Oxford, is also bequeathed £1,000. To the Manchester Lock Hospital, the Hospital for Incurables, the Clinical Hospital, and the Pendlebury Hospital for Sick Children are allotted £500 each. Of London charities, the Brompton Cancer Hospital, the Royal Hospital for Incurables, Great Ormond Street Children's Hospital, the National Paralytic and Epileptic Hospital, and the Idiot Asylum, Earlswood, each receive £500.

On Saturday last the House Committee and a number of the Governors of the Morley House Convalescent Home paid a visit to the present establishment at St. Margaret's Bay, Dover, and held a meeting to consider the question of the demolishment of the old building and the erection of a more suitable and commodious one. A large wing, it will be remembered, was opened in September, 1893, for the additional accommodation of fifty patients. This would, therefore, afford plenty of room for patients during the winter, while the rebuilding of the rest is being carried on. Mr. G. D. Stevenson, the hon. architect, submitted plans, the cost of which he estimated at about £3,000. For this sum the committee resolved to make an appeal at once, in order that the work may be put in hand without delay, and the new building be ready for the reception of patients early in next year.

Our contemporary, *The Gentlewoman*, is offering £200 in Christmas gifts to charities to be selected by its readers. The metropolitan charities are excluded from these gifts, which are confined to Ireland, Wales, and Scotland, and two English counties. The proprietors state that they have merely a general knowledge of the chief charitable institutions, and they therefore desire the readers of the paper to select the charities. The results of the voting will be published week by week so as to stimulate interest in the competition. Whilst we rejoice that £200 is to be given to the charities, we feel that the plan adopted is not likely to secure any result of practical value. If *The Gentlewoman* confesses comparative ignorance, how can individual readers be expected to possess it? The result should be to secure the gift for the secretary of the charitable institution who is able to stimulate a sufficient number of governors to write to the paper offering the money. The maximum gift—£50—is not a large sum, and it soon goes in postage and stationery; and if every voter has to purchase a copy of *The Gentlewoman* in order to obtain a form of recommendation to fill in, we are inclined to think that our enterprising contemporary, rather than the charities, is likely to reap the greatest benefit from this form of competition. We prefer the plan adopted by *Tit Bits*, which made its distribution according to the ascertained needs and claims of the charities selected to participate.

